# The Elemental Profile of Beer Available on Polish Market: Analysis of the Potential Impact of Type of Packaging Material and Risk Assessment of Consumption

**DOI:** 10.3390/molecules27092962

**Published:** 2022-05-05

**Authors:** Magdalena Gajek, Piotr Wysocki, Aleksandra Pawlaczyk, Łucja Sać, Małgorzata Iwona Szynkowska-Jóźwik

**Affiliations:** Faculty of Chemistry, Institute of General and Ecological Chemistry, Lodz University of Technology, Zeromskiego 116, 90-924 Lodz, Poland; piotr.wysocki@p.lodz.pl (P.W.); aleksandra.pawlaczyk@p.lodz.pl (A.P.); 225306@edu.p.lodz.pl (Ł.S.); malgorzata.szynkowska@p.lodz.pl (M.I.S.-J.)

**Keywords:** beer analysis, risk assessment, can, bottle, elemental analysis, discriminant analysis

## Abstract

Twenty-five elements, including the most essential and toxic metals, were determined in fifty beer samples stored in cans and bottles by Inductively Coupled Plasma Mass Spectrometry (ICP-MS), Inductively Coupled Plasma Optical Emission Spectroscopy (ICP-OES) and Cold Vapor Atomic Absorption Spectroscopy (CVAAS) techniques. The packaging material was analyzed using the Scanning Electron Microscopy with Energy Dispersive Spectroscopy (SEM-EDS) technique. The control of the level of individual metals is necessary, not only to maintain the organoleptic properties of the product, but also to fulfill the standards regarding the permissible maximum concentrations. Metals can originate from different sources, including the brewing water, malt grains, hops, adjuncts, fruits, and spices. They may also come from contamination from the brewery equipment, i.e., vessels and tanks, including beer packing, storing and transporting (kegs, casks, cans). Discriminant analysis revealed that the differentiation of three types of beer (Lager, Ale, Craft) was possible, based on elemental concentrations, for the reduced data set after their selection using the Kruskal-Wallis test. The analysis of the impact of the packaging material (can or bottle) proved that when this parameter was used as a differentiating criterion, the difference in the content of Na, Al, Cu and Mn can be indicated. The risk assessment analysis showed that the consumption of beer in a moderate quantity did not have any adverse effect in terms of the selected element concentrations, besides Al. However, in the case of Al, the risk related to consumption can be considered, but only for the beer stored in cans produced from aluminum.

## 1. Introduction

When asked about the most popular alcohol in the world, we can give at least a few correct answers. Certainly, when we consider all drinks containing alcohol, beer will be in the first place, both in terms of production and consumption. According to the data provided by Statista.com, in 2019, as much as 1.91 billion hectoliters of hoppy beverage were produced on our planet. The worldwide sales value of US$587 billion in 2019 is expected to increase to US$867 billion by 2025. Due to the fact that there is a constant and comparable increase in the consumption of beer at home and outside the home, revenues are mostly driven by a rebound in volume consumption and a continuous premiumization of the market. When it comes to the annual consumption of beer per capita, European countries are in the top places all over the world. The country that tops the list is the Czech Republic, with 143.3 L consumed per capita. However, the top five largest beer consuming countries in the world (>100 L per capita) include Austria, Germany and Poland [1].

According to the definition, beer is a product of yeast alcoholic fermentation of extracts of malted cereals, usually barley malts, and flavored with hops and the like for a slightly bitter taste. Beer typically contains less than 5% alcohol [2]. From the point of view of the chemical composition, beer is a drink composed of various organic ingredients, such as proteins, sugars, polyphenols, and amino acids. They are recognized as the by-products of yeast metabolism in the fermentation process and are responsible for most of the organoleptic characteristics of beer [3].

All natural ingredients used for brewing, including water, cereals, barleys and yeasts, are the main source of metals in beer. Therefore, the mineral composition of beer clearly reflects the composition of components used for brewing and relates to processes of its production. However, the content of metals is not constant. It depends on the quality of ingredients taken, the type of beer, and the country of origin. Metals in beer could also come from other substances added during brewing to maintain optimal fermentation conditions and maturation processes. Other external sources of metals in beer could be contamination from brewery equipment, i.e., vessels and tanks, including those used for beer packing, storing and transporting (kegs, casks, cans). In the context of the mentioned packaging material, currently as much as 98% of food products that reach the consumer market require packaging. The materials in which food products are packed (cans, bottles, plastic bags, etc.) are crucial in order to ensure the safety, and maintain the nutritional properties, of food products. According to regulations, materials designed to have contact with food must be safe, their components cannot migrate into food, cannot cause changes in its composition or adversely affect the organoleptic properties of food products. In all European countries and in Poland, the safety of food packaging is regulated by relevant regulations. Packaging intended for contact with food must comply with the provisions of Regulation (EU) No 10/2011 of 14 January 2011, on plastic materials and products designed for contact with food [4]. Despite the regulations being introduced as early as 1996, it was noticed that the poor quality of beer cans resulted in a higher cadmium content in alcohol stored in this type of packaging, compared to beer from barrels [5]. In later years, research also focused on the topic of aluminum cans, kegs and barrels as beer packaging material, from which, under certain conditions, an increased migration of metal ions (e.g., Co, Cr, Fe, Cu, Ni, Al) may occur [6,7]. It is assumed that the longer the beer is stored, the higher the concentration of metals in the beer. Additionally, rapid corrosion can be influenced by storage temperature, which takes place regardless of its coating, and, consequently, the higher the accumulation of metals in beer [6]. From the quality perspective of beer, both the final product and raw materials from individual production stages are tested against pH value. This parameter has a crucial impact on the taste, prevents the growth of microorganisms and is a key factor for beer aging, its stability, and durability [8,9].

A brief and concise literature review included in Table 1 [3,7,10,11,12,13,14,15,16,17,18,19,20,21,22] shows some selected papers in which the content of chosen metals in beer was determined. As can be seen, the most commonly applied techniques for elemental composition are the ones based on ICP as a source of excitation and ionization [3,7,10,11,14,15,16,17,18,20,22] and these use atomic absorption phenomena [12,13,19,22]. Despite the availability of several scientific publications dealing with the elemental analysis of beer, this issue is still not as widely and readily discussed as in the case of wine studies. It is noteworthy that there is a variety of preparatory procedures used by the researchers. Different methods were used in almost every paper cited in the review.

The undeniable benefit of applying chemometric tools is noteworthy. The multivariate methods of data support the performance of instrumental studies, including elemental analysis, influence potential discrimination or differentiation among samples. The latest literature reports clearly show that the use of chemometric tests in the elemental analysis of food allows, among other things, distinction of botanical species of spices from one another [23], or their botanical origin [24]. Moreover, the employment of PCA analysis made it possible to discriminate dried figs according to their geographical origin [25]. Statistical tests and chemometric analysis were also applied to evaluate the results of the elemental analysis of alcohols, including beers, in order to differentiate the tested samples in reference to the type of beer [14] and geographical origin [3].

In this work, elemental characterization of beer available on the Polish market was carried out. Additionally, pH values of each sample were determined. What is more, an assessment of the potential influence of the packaging material on the metal content of the collected beer samples was carried out. The study was undertaken to verify if the packaging material can transfer ingredients into food in amounts that could be life-threatening, or change the composition of beer and, consequently, whether it is safe. In the context of the risk assessment connected with beer consumption in this study, all the calculations were made for 70 kg body weight and 0.5 L average beer consumption. The performed elemental analysis was also used to compare and categorize the analyzed samples with regards to selected parameters (type of beer, type of packaging).

## 2. Results and Discussion

### 2.1. Level of Metals in Analyzed Beer Samples

In this study, the level of 25 elements in 50 beer samples (including three samples of home-made products) were determined. The concentrations of Ag, Cd, Co, Mn, Mo, Ni, Pb, Sb, Sn and Tl were measured by the ICP-MS technique. The ICP-OES technique was applied to assess Al, Ba, Ca, Cr, Cu, Fe, K, Mg, Na, P, S, Sr, Ti and Zn content. The total mercury content was analyzed by the CVAAS technique. Due the fact that in all analyzed samples the levels of Tl and Hg were below the limit of quantification (LOQ), these elements were removed from further steps of data processing. For the remaining elements, only the results for single samples were below the limit of quantification. Thus, the presence of Cu and Fe were not indicated in one sample, Sb and Cr were not determined in three samples, Ag and Pb were not determined in four samples. The presence of Zn was not measured in 22 samples, Al in 35, Ti in 41 and Ba in 44 samples.

Based on the Shapiro-Wilks test (*n* < 100), the hypothesis of a normal distribution for all analyzed variables was rejected. Therefore, a nonparametric test—the Kruskal–Wallis test—was used to analyze the data set. The basic statistical information about the studied variables is given in Table 2.

In the tested beer samples, most of the measured elements showed impressively high compliance with the works of other researchers (e.g., Ba, Ca, Cu, K, Mg and Na [10,16]; Cd, Zn and Cr [11]; Sb, Sn [21]; Ni, Mo [14,15]; Pb, Ti and Ag [22]; P [3]; S [21]; Fe [13]; Mn [10,13,14,16,17]). When it comes to the concentration range for Co, compared to three other studies [15,17,22], in this work, slightly higher values were obtained. In turn, in the work of Pires, L. et al., 2019 [16], the range of cobalt content in seven samples of Brazilian beer were even higher. In this study, craft beer can be in general characterized by higher Co and Sr content. The same trend applies to strontium content. Home-made beer presented the highest content of Sr compared to the other groups. These results significantly exceed the maximum value, so that the range of Sr concentrations obtained in this study is slightly higher compared to the data found in the literature [14,16]. The last element for which the maximum value was measured in this paper is aluminum. However, it should be noted that this metal was only determined in the samples of products derived exclusively from cans. From the remaining samples, this value was below the limit of quantification. Thus, the higher content of a given element was determined by the specific characteristics or properties of the samples, and is not a characteristic value.

Many factors have an influence on the obtained measurement results of individual elements, including the species of raw materials used, soil properties, and agricultural technologies. Apart from the factors arising from raw materials usage, water employed during production could have an impact on elemental characterization. Therefore, three samples of water (from a deep well) used for the production of home beer were analyzed. The water samples were diluted ten times and directly measured in the same way as the studied samples. The dispersion of outcomes within the aforementioned group of water samples was small (RSD values for particular elements did not exceed 1%). The authors in their previous paper [22] included the results of tap water analysis which can be the main source during home-made cider production. The authors can confirm that the biggest differences in the levels of studied elements were connected with concentrations of Ca, Na, Sr or Mg. Based on the obtained results, it can be assumed that the macro-elements (especially Ca, Na, Mg) and strontium contained in the water used for the production of beer have the greatest impact. Despite the fact that home-made beer was additionally characterized by higher values of Cd and Pb compared to other beer samples, and taking into account the results obtained for the water used in their production, it is difficult to see any impact here. Probably the increased values of these elements come from the impurities in the apparatus or other raw materials used in their production. In most cases, the content of the determined metals in the deep well water was mostly at a trace level or below the limit of quantification.

### 2.2. Analysis of the Potential Impact of Type of the Packaging Material

In order to verify the hypothesis of the potential impact of packaging on the elemental composition of alcohol, five beer brands were selected and purchased, both canned and bottled (H, L, W, Z, ZZB (products derived from the ZZ brand)). The tested set consisted of a total of 30 beer samples (three canned and three bottled samples from each brand). The obtained results for the aforementioned set of samples were compared and divided into two groups: the samples in the can and in the bottle. Additionally, an experiment, which was called “washout”, was carried out (the course of the experiment is described in the Section 3). Moreover, in order to check the elemental composition of the packaging material, all cans and bottles used in the “washout” experiment (12 items) were tested by Scanning Electron Microscopy with the Energy Dispersive Spectroscopy (SEM-EDS) technique. The basic characteristics of the samples used in analysis of the potential impact of packaging and “washout” experiment are presented in Table 3.

Considering the studied set of samples (30) in reference to the type of packaging, the existence of statistically significant differences (based on the Kruskal-Wallis test) in the concentrations of the following elements were found: Mn, Al and Na (with a statistical significance at *p*-level below 0.005).

As shown in Table 4, the greatest differences, both in terms of the mean and the median values, were noted for sodium. Rather expectedly, higher values of this element were measured in beer samples stored in glass bottles. Another element having a concentration which is clearly different for samples kept in glass bottles and in cans is aluminum. In the case of glass containers, for all analyzed samples, Al concentration was determined below the limit of quantification (LOQ). On the other hand, both the mean and the median values of Al content in the samples from cans were close to 10 mg/L. The other element for which statistically significant differences were found is manganese. Higher concentration of this element (mean and median values) was recorded for beer samples stored in cans.

In the case of the measured pH value for a set of canned and bottled samples, no statistically significant differences were noted. However, higher pH values were reached by the samples stored in bottles (mean pH 4.62) compared to the samples stored in cans (mean pH 4.51).

In the second part of the analysis (“washout” experiment), the non-parametric Kruskal-Wallis test showed statistically significant differences in the concentration of aluminum only, with a statistical significance at *p*-level below 0.05 (Table 5). However, it should be noted that for the majority of the elements which were determined, their concentrations were below the limit of quantification. Nevertheless, a two-week contact of demineralized water with pH close to the value typical of beer with the packaging material (can) affected the release of Al in the range from 0.040 to 0.550 mg/L.

**Figure 1:**
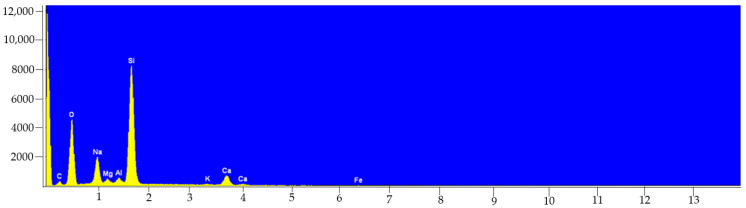
EDS spectrum collected from the surface of glass bottle sample (×100).

The last step in the verification of the potential influence of the packaging material on the elemental composition of beer was the analysis of the cans and bottles in which the alcohol was stored by the SEM-EDS technique. The study aimed to check the real elemental composition of the cans and bottles in which beer is sold. For this purpose, the material of nine bottles (three brands of beer, three items of each) and three cans (three items of one brand) were tested. Despite the use of glass bottles in three different colors (transparent, brown, green) in this study, the obtained EDS spectra did not differ significantly from each other. In the exemplary EDS spectrum presented in Figure 1, the peak with the highest intensity belongs to the main component used in the production of glass, i.e., silicon (present in the glass in the form of SiO_2_). The remaining elements that were identified and can be related to the composition of the glass include: Na, Mg, K, Ca, Fe.

**Figure 2:**
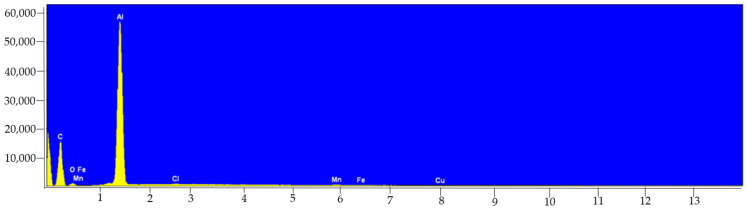
EDS spectrum collected from the surface of aluminum can sample (×100).

In turn, in the case of the analysis of beer cans, Al can be indicated as the basic component, but also peaks originating from Mn or Cu were visible (Figure 2). In the case of this study, no differences in the spectra between the three tested objects were found. However, it should be noted that all cans were from the same manufacturer.

Indeed, literature reports suggest that the level of pH values (~4.2) has a significant impact on the content of metal ion ingestion (e.g., Co, Cr, Fe, Cu, Ni, Al), especially in the case of aluminum cans or kegs [6,7]. In the case of Al, it can be suspected that the longer beer is stored, the higher the beer content of this metal will be. It was also noted that a higher storage temperature influences the corrosion rate of the inner layer of the can, which promotes the release and accumulation of ions from the packaging material into beer [6]. The results obtained in this study are therefore in line with quoted literature reports. The element which was released from the cans the most certainly is aluminum. This was proven by the experiment of “washout” (Table 5). This is not surprising as Al is the basic component of food storage cans. It should be emphasized that in order to strengthen resistance to crushing, clean aluminum is combined with harder metals, such as copper or manganese as alloys. This guarantees the reduction of the weight of the structure [26]. Therefore, the presence of elements, such as Mn and Cu, in much higher concentrations in canned products is directly related to the composition of the alloy (Table 4 and Figure 2).

Recent literature data suggest that there is clear evidence of a correlation between the type of bottle in which drink is stored and the release of various components, including metallic contaminants. Reimann et al., 2010 [27], compared the content of 57 elements in 294 samples of water from the same producers which were stored in glass, as well as in PET bottles, by the ICP-MS technique. The cited study showed higher concentrations of some elements in glass bottles when compared with PET bottles (e.g., Sb, Pb, Zr, Cu, Al, Fe, Ti, Zn, Cr and Sn). Also, the investigations of the influence of the color of glass bottles confirmed that water kept in green glass bottles had significantly higher concentration of elements such as Cr, Ti, Fe and Co. Gajek et al., 2021 [22], stated that the level of sodium turned out to be decisive in discriminating cider samples with regards to the packaging in which the cider was stored, proving that the level of this element was much higher in products from glass bottles. Again, this fact is related to the components used to produce this type of packaging material. Glass sand accounts for about 75% of the glass used in the food industry (mainly in the form of silicon dioxide). In turn, sodium oxide (less often potassium oxide) accounts for about 12% of the total weight of a glass bottle [28,29]. Hence, the much higher concentration of sodium in products from glass bottles is precisely associated with the composition of this type of packaging (Table 4, Figure 1). During the process of sample preparation for analysis (mineralization and dilution), alcohol samples did not come into contact with other glass vessels that could be the source of Na.

#### Analysis of the Impact of Packaging Material after Taking into Account Beer Brand

In the last step of the analysis dedicated to the evaluation of the impact of packaging on the concentration of elements in beer samples, an additional parameter was introduced, i.e., the beer brand. The analyzed set of samples, including both canned and bottled products (30 samples), contained five independent brands of beer (H, L, W, Z, ZZ(ZZB)). Considering the studied set of samples in reference to the brand, the existence of statistically significant differences (based on the Kruskal-Wallis test) in the concentration of studied elements was confirmed for the variables included in Table 6. For these metals the level of significance (*p*) for considered pairs was less than 0.05.

**Figure 3:**
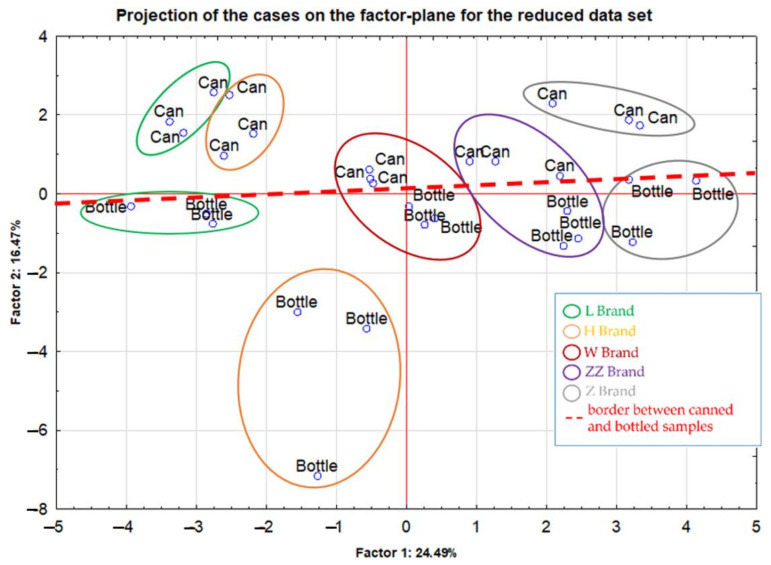
Projection of the cases on the factor-plane for the reduced data set.

Projection of the cases on the factor-plane which was performed clearly revealed the possibility of distinguishing between products stored in cans and bottles, since the division of these samples can be observed along the red dotted line used as a border in Figure 3. Almost all canned samples were located at the top of the plot (first and second quadrants). In turn, the lower part of the graph (third and fourth quadrants) was occupied only by samples stored in glass bottles. Moreover, apart from the impact of the packaging material parameter, in some cases it was possible to group the analyzed samples according to their brand. The strongest compliance within a given brand was recorded in the case of objects belonging to the ZZB brand (marked in violet) and W (marked in red). The H brand (marked in orange) was characterized by the greatest dispersion of results and separation of the samples from the can and bottle. But it should be noted that all the brands mentioned so far (ZZB, W and H) originated from a common manufacturer. Interestingly, the brands which are situated in the central part of the projection plot are produced in independent breweries. Moreover, for the ZZB brand statistically significant differences for the same elements (Co, Ag and Cd), as compared to the H and L brands, were reported. Beer samples from the other two brands, L (marked in green) and Z (marked in grey), were located in the most extreme positions in the presented projection diagram (Figure 3). In this case, the influence of an additional parameter, which was the packaging material, seems to be more crucial, especially in terms of dispersion on the objects within this brand, and was much higher in the case of the W and ZZB brands.

Certainly, some spread of the results within the mentioned brands, especially in the case of the H brand beer samples stored in cans, may also result from the fact that the products were purchased at time intervals and that they might originate from different production batches.

**Figure 4:**
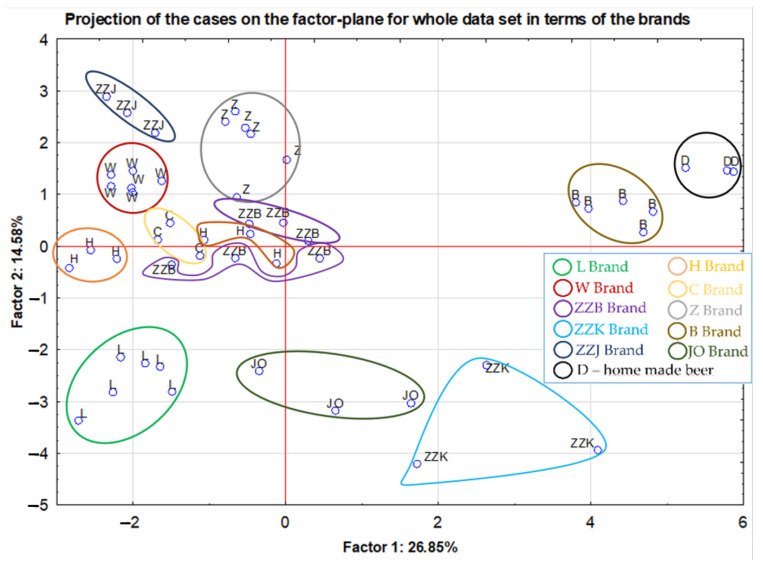
Projection of the cases on the factor-plane for whole data set in terms of the brands.

Taking into account two parameters (type of packaging material and brand) for the reduced set of samples, the PCA method allowed only for the selected brands to be distinguished. For this reason, the possibility of differentiating in terms of brand of all analyzed beers was checked. Thus, 50 samples from 11 brands were tested (where ZZB, ZZK and ZZJ were brands originated from the joint producer ZZ). As was revealed by the performed projection of the cases in the factor-plane (Figure 4), for most of the studied groups it was possible to separate the analyzed samples according to their brand. Again, the locations of the samples belonging to the W brand (marked in red) were grouped into one cluster. The samples belonging to the L (marked in green) and Z (marked in grey) brands this time formed one common cluster, where the influence of the packaging material was much less visible than in the plot above (Figure 3). In the central part of the graph, there were beer samples belonging to the C brand (marked in yellow), stored only in glass bottles. The brands JO (marked in dark green), B (marked in brown) (JO and B both are craft beer samples) and D (marked in black; home-made beer) were very distinct from each other and separated from the rest of the clusters. However, for the brand JO, the inconsiderable dispersion of results for three samples taken from independent bottles was observed. The explanation for this phenomenon is certainly the fact that each of these beer samples had different aromatic and flavor additives. As in the case of the previous analysis, a clear separation of the samples from the H company (marked in orange) was observed. Again, it could be related to different packaging material. On the right side of the graph samples stored in cans were situated, while in the center of the graph samples stored in bottles were placed. As part of the manufacturer ZZ, the brands ZZB (marked in violet), ZZJ (marked in navy blue) and ZZK (marked in blue) were separated. As the graph below shows, each of these brands was grouped together, but in different regions on the projection plot. It indicates that the objects belonging to ZZJ and ZZK brands occupied the most extreme positions on the graph (second and fourth quadrants). This is due to the fact that they represented completely different types of beer, Ale and Lager’ where the first one is a result of bottom fermentation, while the second one is from top fermentation. On the other hand, within the group of samples originating from the last of the mentioned brands, i.e., ZZB, despite a similar position on the plot, a clear separation between the products stored in cans and those from bottles was found.

### 2.3. Differentiation of the Analyzed Beer Type According to Their Element Contents

In this study, three groups were distinguished among the analyzed samples: Ale (top-fermented beer—9 samples), Lager (bottom-fermented beer—30 samples) and craft beer (11 samples). Due to the lack of data on the type of fermentation for beer from small artisanal breweries, this type of sample was classified separately as craft beer. In the first step, the existence of statistically significant differences between the analyzed groups was checked (Table 7). The level of significance (*p*) for considered pairs was less than 0.05. An extremely interesting relationship that was observed is the fact that in as many as 7 out of 10 parameters (Co, Ni, Ag, Cd, Pb, Cu and Zn) for which statistically significant differences were noted, the median values were arranged in the following order: Lager < Ale < Craft. Only for the median value of Ca, this tendency was exactly the opposite: Craft < Ale < Lager. In turn, the median Mn concentration and pH values are the highest for the Ale beer type. Thus, the Kruskal-Wallis test was used as the first approach to exclude variables without discriminant power [30] and, therefore, these elements were not considered for further analysis.

In order to eliminate the potential influence of additional factors on the differentiation of beer according to their type, a set of cases was reduced from 50 to 38. Only samples with the same type of packaging (bottle) were included in the new tested data set. In this case, statistically significant differences were reported for such parameters as Ag, Ca, Cd, Co, Ni, Pb, Zn and pH (*p*-value less than 0.05). Therefore, in relation to the former comparison of all beer samples (50 objects), no statistically significant differences for Mn and Cu were stated. This allows us to strengthen the hypothesis that these elements (Mn, Cu), as indicated in the previous section, were closely related to the type of packaging parameter, and as a consequence of the rejection of samples from cans the influence of this factor was eliminated.

In the next part of the data evaluation, PCA analysis was performed. For the whole data set, after taking into account the first eight components, over 80% of the explained variance was obtained. However, after taking into account the results of the variables after reduction, for the first two components, almost 79.11% of the explained variance was reached (80% of the explained variance was obtained for the first three components). Assuming the Kaiser criterion, two first principal components can be taken into account. Thus, the projection of variables into the factor plane for the reduced set of variables used the following parameters: concentrations of Co, Ni, Ag, Cd, Pb, Ca, Zn, Al and pH values. In this case, the correlation matrix was factored and suitable for PCA (K-M-O test values: 0.740; approximate chi-square value: 427.4 with *p*-level below 0.001).

**Figure 5:**
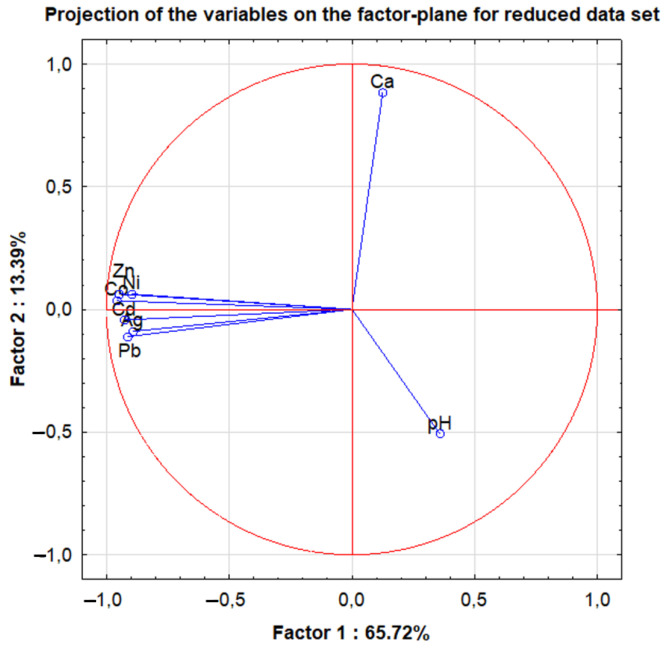
Projection of the variables on the factor-plane for reduced data set.

As shown in Figure 5, Factor 1 has the greatest share in the explained total variance (65.72%). Elements such as Co, Ni, Ag, Cd, Pb and Zn, are most strongly negatively correlated with this component.

**Figure 6:**
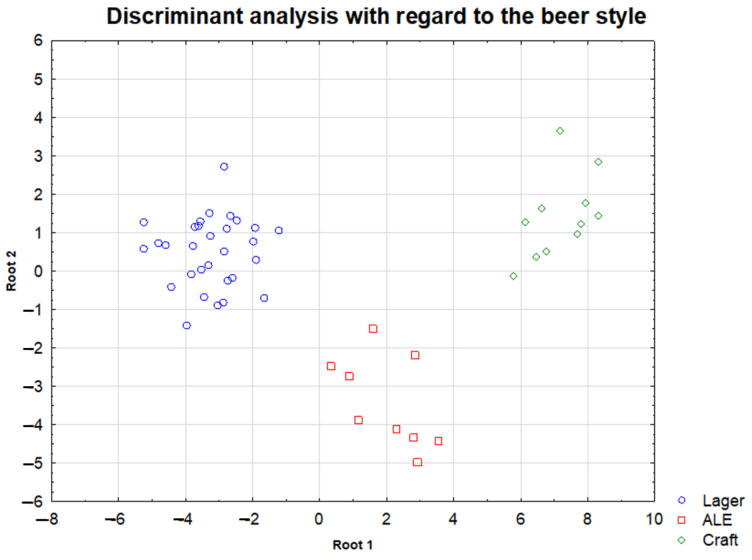
Discriminant analysis with regard to the beer type (Lager, Ale, Craft).

Additionally, discriminant analysis (DA) was performed to evaluate the possibility of distinguishing samples according to the type of beer with a given set of variables. The standard mode of DA was applied to the raw data matrix after dividing the whole data set into three groups (Lager, Ale, Craft). Canonical analysis showed two discriminant functions (DFs), including 24 variables. Both discriminant functions turned out to be significant (canonical correlation R > 0.85; *p*-value < 0.05). The first discriminant function accounted for over 85% of the explained variance, which is equivalent to the discriminant power of this function. The second function explains less than 15% of the total power of discrimination. The means of the canonical variables make it possible to determine the groups that were best distinguished (discriminated against) by each discriminant function. In the first function, the group of craft beer showed the highest canonical variables (6.999) followed by the group of Ale beer (1.471) and Lager beer (−3.008). In the second dimension, the highest canonical variables were determined in the group of Ale beer (−3.555) followed by Lager beer (0.589) and craft (1.302). From the presented data it appears that the first discriminant function distinguishes craft beer from others and the second discriminant function, on the other hand, seems to distinguish Ale-type beer from the rest. As chemical descriptors which are significant for defining the group of craft beer samples Cu and Cd could be indicated. The Ale sample group is best defined by the pH value, in turn the lager group by Cr and Sb. Figure 6 shows the distribution of the samples in the plane of the two obtained DFs. All the samples from the three groups appear separated, so it can be argued that the selected variables are powerful descriptors to characterize beer from the three types of beer.

A similar approach to discriminate beer samples was used by Alexa et al., 2018 [14] and Alcázar et al., 2012 [3]. In the first mentioned paper, 24 samples of local beer, according to its type, were differentiated. The authors distinguished the following 4 types: pale barley, dark barley, pale wheat and dark wheat, and achieved relatively good discrimination between them (especially between the pale barley and dark barley groups) [14]. The study by Alcázar [3] covered beer from Germany (*n* = 15), Portugal (*n* = 18) and Spain (*n* = 35), and the discriminatory analysis in terms of the country of origin was performed after variable reduction. The authors emphasized that this kind of study is of interest due to the current trend in the European Union connected with establishing Protected Geographical Indication of Beer. The parameters indicated by the authors (contents of iron, potassium, phosphorus, phosphate and total polyphenols) turned out to be powerful for their discrimination, as the samples from three different countries were well separated from each other [3].

### 2.4. Risk Assessment

Due to the huge popularity and steadily increasing consumption of beer (in Poland > 100 L per capita), the authors of this publication decided to check the potential risk associated with the consumption of this alcohol. The verification of the risk resulting from the regular consumption of not only beer [14] but also functional drinks [31] has been the subject of recent interest. The literature data clearly indicate that the permissible standards can be exceeded [22,32,33,34]. In the first step, it was checked whether the limits of permissible content of heavy metals in the tested beer samples were met. The authors of this work used an internal national standard that defines the maximum permissible content of selected heavy metals (Cd, Pb) in beer [17]. Since all of the analyzed samples came from Poland, the comparison with the aforementioned standards seems to be correct. The maximum lead content was set at 0.1 mg/L, and cadmium at 0.02 mg/L [35]. However, in regards to this study, the permissible Pb and Cd levels were not exceeded in any case.

In the subsequent step, the risk related to the regular consumption of certain portions of beer was verified.

In order to obtain information on the value of provisional tolerable daily intake (PTDI) for the selected elements, the following data bases were used: WHO JECFA (Joint FAO/WHO Expert Committee on Food Additives), EFSA (European Food Safety Authority), HC1 (Health Canada), SCHER (Scientific Committee on Health and Environmental Risks). These values are shown in Table 8. Risk assessment was performed for the following elements: Mn, Co, Ni, Mo, Ag, Cd, Sn, Sb, Al, Ba, Cr, Cu, Fe, Sr, Zn. PTDI value was withdrawn for Pb, and Tl and Hg concentrations were lower than LOQ in each sample.

Risk assessment of beer consumption in this study was calculated for 70 kg body weight and 0.5 L average beer consumption (Table 9).

Some of the blanks that appear did not contain the analyzed element in higher content than LOQ. According to the given standards, if the value is lower than 1, we can expect risk. If the value is in the range 1–10, risk is possible and above 10 risk is negligible. As in Table 8, besides Al none of the elements could be found in the analyzed samples in concentration which could mean a potential hazard to consumers, because the calculated values of risks were much higher than 10 for all samples. The aluminum concentration, as previously emphasized, was determined in beer samples from cans, and the risk related to consumption can be considered as possible (values ranging from 1 to 10). Perhaps it can be related to the poor quality of aluminum cans dedicated to food storage.

In the study of Alexa et al., 2018 [14], an assessment of the risk associated with moderate consumption of beer was also carried out; however, only for a few elements (Al, Cu, Zn). The authors made the calculations based on two weight-related assumptions, namely 60 kg and 90 kg. Therefore, it can be assumed that the performed calculations concern an average woman and an average man. However, there was no risk to the consumer for any of the samples. It is true that assuming a lower body weight (60 kg), the calculated risk values were lower, but still above 10, so it can be suspected that consumption of 0.5 L beer per day is safe from the point of view of the presence of selected elements.

## 3. Materials and Methods

### 3.1. Samples

Fifty beer samples (35 bottled and 15 canned) of different brands and from various manufacturers were purchased in Łódź Voivodeship (Poland) for analysis. When selecting brands, the authors took into account their popularity and availability on the market. However, in the considered set of samples, there were beer samples that came not only from large national or international companies, but also from local manufacturers. The latter were recognized as craft beer. Additionally, three samples of home-made beer were analyzed. The names of brands are coded, and the manufacturers’ names are not given in this paper. Detailed characteristics of beer samples are summarized in Table 10.

### 3.2. Samples Preparation and Equipment

#### 3.2.1. Beer Sample Digestion

The first step in the preparation procedure of beer samples involved application of an ultrasonic washer (Bandelin Sonorex Digitec, Berlin, Germany). This step was necessary to get CO_2_ out of the beer samples. Then, 4 mL of each analyzed sample were taken with an automatic volumetric pipette, and put into Teflon^®^ test tubes and weighed on an analytical scale. Subsequently, 4 mL of 69–70% HNO_3_ (Baker, Avantor Performance Materials Poland S.A., Gliwice, Poland) were added to each sample in small portions due to the strongly exothermic nature of the reaction. In the next step, microwave mineralization was applied. A detailed description of the parameters of the applied matrix decomposition procedure of alcohol samples was given by Pawlaczyk et al., 2019 [47]. After the mineralization process, the contents of the tubes were quantitatively transferred into Teflon^®^ flasks and diluted to a volume of 25 mL with the addition of a known amount of Certified Material of In used as an internal standard (In; Merck, Warsaw, Poland). The blank samples were prepared in the same way as the studied samples.

#### 3.2.2. ICP-OES and ICP-MS

Measurements of the content of selected elements using spectrometry techniques were carried out on the basis of calibration curves to create a standard solution of CPAchem (Multi-element ICP standard, Stara Zagora, Bulgaria), and six single element standards of In (ICP class, Merck, Darmstadt, Germany), Sb (ICP class, Merck, Darmstadt, Germany), Sn (ICP class, Chem Lab NV, Zedelgem, Belgium), and Ti (ICP class, Radian International LLC, Austin, TX, USA), S (ICP class, Merck, Darmstadt, Germany), P (ICP class, SCP Science, Montreal, QC, Canada). Preparation of standards was carried out by the subsequent dilution method.

Inductively Coupled Plasma Mass Spectrometry with Quadrupole Analyzer ICP-QMS (Thermo Electron Corporation, X SERIES, Rugby, UK) was applied to determine the levels of elements in 50 beer samples for the isotopes ^107^Ag, ^111^Cd, ^59^Co, ^55^Mn, ^95^Mo, ^60^Ni, ^208^Pb, ^121^Sb, ^118^Sn and ^203^Tl. In turn, the contents of Al (396.152), Ba (455.403 nm), Ca (393.366 nm), Cr (267.716 nm), Cu (327.396 nm), Fe (238.204 nm), K (766.490 nm), Mg (279.553 nm), Na (589.592 nm), P (185.942 nm), S (180.731 nm), Sr (407.771 nm), Ti (334.941 nm) and Zn (213.856 nm) were determined by Inductively Coupled Plasma Optical Emission Spectroscopy (ICP-OES) (Thermo Scientific, ICAP 7400 series, Waltham, MA, USA). The basic parameters of operating conditions are summarized in Table 11.

#### 3.2.3. Evaluation of the Correctness of the Obtained Results

For both analytical techniques, for each of the beer samples three replicates were carried out (% RSD was within the range of 0.01–5.00%, even for elements determined at very low levels). Certified Reference Material of TMDA 54.6 (fortified lake water sample by National Water Research Institute, Burlington, Halton, ON, Canada) was used to ensure the quality of analysis. This material did not require any preparation or dilution because the levels of the elements contained therein were at the appropriate levels for this study. For elements not included in the certified TMDA 54.6 material, the measurement correctness was verified on the basis of Certified Reference Material of human hair (NCS ZC 8100 2b). The measured concentrations of individual elements in the CRM agree well with the certified values and can be proven by the recovery values. The certified value of individual elements in CRM and the obtained values are in Table 11. The analogous procedure of assessing the accuracy of the proposed method was described previously by Gajek et al., 2021 [22].

The coefficient of the linear regression for each analyte was from 0.999 to 1.000. Sensitivity of the applied method was examined as the limit of detection (LOD) and limit of quantification (LOQ). Values of the standard deviation of the results obtained for a series of blank samples were the basis for setting the above-mentioned limits, and they were deduced from mathematical expressions: LOD = x_śr_∙3SD and LOQ = 3 ∙ LOD [48]. The obtained outcomes are presented in Table 12.

#### 3.2.4. CVAAS

In this work, an automatic mercury analyzer MA-3000 (Nippon Instruments Corporation, Tokyo, Japan) was applied to evaluate the total mercury content in 50 beer samples. The measurement procedure was the same as the one extensively described by Pawlaczyk et al., 2019 [47].

#### 3.2.5. SEM-EDS

In order to determine the elemental composition of tested can and bottle material, a scanning electron microscope SEM (HITACHI S-4700, Kagawa, Japan) with an energy dispersive X-ray spectroscopy EDS (Thermo Scientific NORAN System, Waltham, MA, USA) was used.

The essence of the microscope’s operation is the interaction of the electron beam with the sample, which results in the formation of low-energy secondary electrons (SE), high-energy backscattered electrons (BSE) and the emission of X-rays. The use of the X-ray microanalysis (EDS) attachment allows the performing of semi-quantitative elemental analysis of the surface of the test sample.

What is important to note is that the tested object should not have magnetic properties. Therefore, before starting the research, it was necessary to check possible magnetic properties.

For scanning electron microscopy analysis, the samples were placed on a carbon plaster. Analyzed samples did not require any coating. As part of the research under the scanning electron microscope, the EDS spectra of elements from the sample surface were collected.

### 3.3. “Washout” Experiment

The “Washout” experiment consisted of the collection of packaging material (9 bottles including 3 green (H), 3 brown (W), 3 white (ZZB) and 3 cans (Z)) designed to store the beer samples. The packaging material was washed, rinsed with demineralized water, and fulfilled with 50 mL of demineralized water with the addition of nitric acid in order to obtain the appropriate pH (~4.5) and left covered for a period of 2 weeks. After this time, the contents of the above-mentioned packages were tested.

### 3.4. Risk Assessment

The following equation (Equation (1)) was used to estimate the risk associated with moderate consumption of beer:

(1) $$Risk=\frac{TDI}{ADI}=\frac{PTDI\cdot bw}{C_{element}\cdot dc}$$

*TDI*—tolerable daily intake

*ADI*—average daily intake

*PTDI*—provisional tolerable daily intake

*bw—*body weight

*C_element_—*element concentration

*dc*—daily consumption

### 3.5. Data Analysis

All analytical measurements were carried out in triplicates. The obtained outcomes were described using basic statistics: mean, median, minimum and maximum values. The quantitative data were expressed as the box and whisker plots, with a median value chosen as a central value. The Shapiro-Wilks test (N < 100) was used to check normality of distribution. The hypothesis about a normal distribution for all analyzed variables was rejected. The Kruskal-Wallis non-parametric test was used to evaluate the significance of differences in the measured levels of variables among particular groups according to the considered parameters, such as the type of packaging and the type of beer. To increase the interpretability of the outcomes, Principal Component Analysis (PCA) and Discriminant Analysis (DA) were performed.

For the statistical and multivariate analysis, the STATISTICA 12.5 (New York, NY, USA) software was used.

## 4. Conclusions

In this study, the content of 25 micro and trace elements in 50 beer samples entirely produced in Poland from 9 different producers were measured to check possible relations between types of beer (Lager, Ale, Craft), type of packaging (can, bottle), and elemental concentrations.

The analysis of the impact of the packaging material showed that the products stored in aluminum cans were characterized by higher Al contents compared to the products stored in glass bottles. The hypothesis regarding metal permeation, which is the main component of the packaging material for alcoholic beverages, was also confirmed by the “washout” experiment. Additionally, the existence of statistically significant differences in the concentrations of Cu and Mn (components linked with the composition of the aluminum alloy to ensure resistance to crushing) between canned and bottled beer were shown. On the other hand, beer stored in glass bottles was characterized by higher Na content.

In the context of distinguishing the analyzed beer samples according to their type (Lager, Ale, Craft), the existence of statistically significant differences between the studied groups was shown in the concentrations of the following elements: Co, Ni, Ag, Cd, Pb, Cu and Zn as well as in pH values. It is interesting that the median values for most elements were arranged in accordance with the following order: Lager < Ale < Craft. The number of input variables was reduced according to the results of the Kruskal-Wallis test. Thus, in the discriminant analysis, the differentiation of these three types of beer samples could be possible, based on element concentrations for the reduced data set of variables (clear separation of the groups being compared).

The risk assessment analysis showed that moderate beer consumption does not have any adverse effect in terms of the selected element concentrations. Besides Al, none of the analyzed elements was present in the studied samples in hazardous concentration. However, in the case of Al, the risk related to consumption can be considered, but only for beer stored in cans produced from aluminum.

## Figures and Tables

**Table 1:** A literature review on the content of metals in beer samples.

Samples	Elements	Technique + Preparation Process	Conclusions	Ref.
68 samples of beer (15 samples from Germany, 18 samples from Portugal, 35 samples from Spain)	Al, Ba, Ca, Fe, K, Mg, Mn, Na, P, Sr, Zn	ICP-AES Digestion with 65% HNO_3_ and 30% H_2_O_2_	Elemental contents for all samples [mg/L]: Al: 0.05–2.78; Ba: 0.01–0.06 Ca: 21.8–108.5; Fe: 0.03–1.41 K: 251.0–570.3; Mg: 43.1–210.4 Mn: 0.03–0.35; Na: 8.4–129.6 P: 108.6–382.3; Sr: 0.10–0.76 Zn: n.d.–0.98	[3]
35 samples of Polish beer (30 bottled and 5 canned)	Ag, Cd, Co, Cr, Cu, Fe, Hg, Mn, Ni, Pb, Sb, Sn, Zn	ICP-MS Digestion of 1 mL of degassed beer with 5 mL of concentrated HNO_3_ in automatic microwave digestion system	Elemental contents for all samples [ng/mL]: Ag: 0.01–0.20; Cd: 0.02–0.53 Co: 0.08–0.57; Cr: 3.8–45; Cu: 29–150; Fe: 45–530; Hg: 0.08–0.64; Mn: 53–470 Ni: 3.8–200; Pb: 1.4–6.0; Sb: 0.16–0.91; Sn: 0.42–3.4; Zn: 4.0–120	[7]
8 samples (4 beer samples and 4 wort samples)	Ca, Cu, Fe, K, Mg, Mn, Na, Zn	ICP-OES Degasification (20 min) + filtering (wort samples only) + dilution of 8 mL wort sample in 32 mL of 8.8% HNO_3_ and 3.8% ethanol; dilution of 10 mL beer sample in 10 mL of 14% HNO_3._ All samples were stored at 4 °C before analysis	Metal contents for wort samples [mg/L]: Ca: 74–96; Cu: 0.195–0.357 Fe: 0.045–0.149; K: 775–1125 Mg: 111–178 mg/L; Mn: 0.150–0.263 Na: 26–36; Zn: 0.163–0.262 Metal contents for beer samples [mg/L]: Ca: 61–101; Cu: 0.047–0.148 Fe: 0.023–0.038; K: 464–850 Mg: 90–145; Mn: 0.132–0.308 Na: 12–26; Zn: 0.000–0.028	[10]
48 samples of beer (16 samples from following stages of the production of 3 types of lager craft beer from 4 breweries)	As, Cd, Cr, Cu, Fe, Ni, Pb, Zn	ICP-MS Microwave mineralization with 3 mL of 65% HNO_3_, 1 mL of 30% H_2_O_2_ and 0.2 mL of 50% HF	All samples were measured in four breweries. Metal contents [μg/kg]: Cd: n.d.–91.43 Cr: n.d.–118.15 Cu: n.d.–3723.10 Fe: n.d.–91.05 Ni: <LOD for all samples Pb: <LOD for all samples Zn: n.d.–1081.60	[11]
4 samples of Brazilian beer	Cu, Mn, Pb, Zn	TS-FF-AAS, GFAAS Degasification in an ultrasonic bath for 20 min + dilution in the proportions 1:1 to 1:5 with 0.14 mol/l HNO_3_ depending on the analyte and the sample	Metal contents for all samples [μg/L]: Cu: TS-FF-AAS: 38.0–155; GFAAS: 39.9–160 Mn: TS-FF-AAS: 110–348; GFAAS: 117–355 Pb: TS-FF-AAS: 13.0–32.9; GFAAS: 14.1–4.6 Zn TS-FF-AAS: 52.7–226; GFAAS: 55.3–231	[12]
6 samples of Polish beer	Cu, Fe, Mn	F-AAS Filtration + degasification of 20 mL of the sample + digestion on a hot plate in glass beakers to reduce the sample volume to about 1 mL + dilution with 3 mL of concentrated HNO_3_ and evaporation of the samples nearly to dryness. Addition of 5 mL of 30% H_2_O_2_ to the residues + heating. Reduction of volume up to 1 mL and subsequent dilution to 10 mL.	Metal contents for all samples: Cu: 0.072–0.114 mg/L Fe: 0.209–0.345 mg/L Mn: 0.070–0.165 mg/L	[13]
24 samples of 4 types of beer (pale barley, dark barley, pale wheat and dark wheat) Samples from 5 countries	Al, Ba, Cd, Co, Cr, Cu, Fe, Mn, Mo, Ni, Pb, Sr, Zn	ICP-MS Degasification + 20 mL of beer sample + 5 mL of HNO_3_ + left to stand overnight. Addition of 5 mL of HNO_3_ to beer sample at a speed of 1 mL/h + pre-digestion at temperature 60 °C for 30 min. Addition 3 mL of 30% H_2_O_2_ + digestion at 120 °C for 90 min + dilution to final volume of 50 mL with ultrapure water	Metal contents for all samples [μg/L]: Al: <LOD–92.8; Ba: 9.95–39.9 Cd: <LOD; Co: 0.169–0.481 Cr: 0.919–16.7; Cu: 27.3–109.0 Fe: <LOD; Mn: 41.0–260.0 Mo: 1.73–18.3; Ni: <LOD–13.7 Sr: 50.7–212.0; Pb: <LOD–6.01 Zn: 23.9–101.3	[14]
40 samples of beer (30 bottled and 10 canned)	Cd, Co, Cr, Mo, Ni, Pb, Sb	ICP-MS Placement of 4.50 g of beer sample into a Teflon beaker + drying on a hot plate at 185 °C. Addition of 3 mL of concentrated HNO_3_ + the heating of the sample at 185 °C until only a few drops were left. Addition of 1 mL of 30% H_2_O_2_ + heating of the sample until it is dry + cooling the beaker. Addition of 2% HNO_3_ + dilution to final volume of 10 ml	Metal contents for all samples: Cd: n.d.–2.63 ng/g Co: 0.08–0.48 ng/g Cr: 2.97–40.61 ng/g Mo: 0.74–29.05 ng/g Ni: 2.20–26.76 ng/g Pb: 0.23–7.48 ng/g Sb: 0.11–1.97 ng/g	[15]
7 samples of Brazilian beer (Pilsen, craft and dark)	Al, Ba, Ca, Co, Cr, Cu, Fe, K, Mg, Mn, Na, Ni, Sr, Zn	ICP-OES, MIP-OES Dilution of the samples with 1.0 M HNO_3_ at ratio 1:4 with internal standard solutions (Be, Ga, Sc, In and Y)	Metal contents for all samples measured by ICP-OES [μg/L]: Al: <LOQ–401; Ba: 6–154; Ca: 12–112; Co: 95–195; Cr: 30–37; Cu: 30–81; Mn: 88–160; Fe: <LOQ–86; Ni: 318–610; Sr: 53–475; Zn: 55–164 [mg/L] K: 251–548; Mg: 24–79; Na: 51–171	[16]
30 samples of Greek beer (11 Ale, 10 Lager, 5 Pilsner, 2 Porter, 1 Weiss and 1 Barley wine beer)	Ba, Cd, Co, Cr, Cu, Fe, Mn, Ni, Pb, Sr, Zn	ICP-MS Ultrasonication with N_2_ + digestion with 65% HNO_3_ and 30% H_2_O_2_	Mean values of metal contents in beer [μg/L]: Ba: 11–56; Cd: 0.01–1; Co: 0.001–1.1 Cr: 1.7–48; Cu: 0.01–84; Fe: 58–838 Mn: 44–377; Ni: 3.1–40; Pb: 0.39–11 Sr: 58–292; Zn: 0.05–105	[17]
2 samples of beer (brewed from 100% malted black and white rice from India)	Ag, Al, Ba, Ca, Cd, Co, Cr, Cu, Fe, K, Mg, Mn, Mo, Na, Ni, P, Pb, S, Sr, Ti, Zn	ICP-MS Degasification (5 min) + dilution with 2% HNO_3_ at ratio 1:10	Higher contents of the following elements in white rice beer: Cd; Co; Cr; Cu; Fe; Mo; Na; Ni; Pb; S; Sr Higher contents of the following elements in black rice beer: Al; Ba; Ca; K; Mg; Mn; P; Ti; Zn	[18]
20 samples of beer (13 from Brazil and 7 imported)	Al, Cd, Cu, Pb	GFAAS Degasification of beer + dilution with 0.2% HNO_3_	Mean contents for all samples [μg/L]: Al: 10.0; Cd: 0.6; Cu: 17.0; Pb: 29.0	[19]
20 samples of Romanian bottled beer	Al, Ba, Ca, Cd, Cr, Cu, Fe, K, Mg, Mn, Na, Ni, P, Pb, Zn	ICP-MS, IRMS Degasification of 20 mL of beer for 20 min + filtration by passing through a 0,45 μm pore size membrane filter (process was repeated 3 times for complete degasification)	Elemental contents for all samples measured by ICP-MS [μg/L]: Al: 64.6–2617.6; Ba: 5.2–75.7 Cd: <0.001–0.4; Cr: 20.3–441.7 Cu: 25.9–73.6; Mn: 4.2–231.7 Ni: <0.001–187.1; Pb: <0.001–12.6 Zn: <0.001–704.2 [mg/L]: Fe: 0.2–3.5; Ca: 7.8–62.2; K: 29.8–197.0 Mg: 22.5–84.7; Na: 5.2–154.9; P: 66.0–154.3	[20]
30 samples of Brazilian beer	Ca, Cu, Fe, K, Mn, Ni, P, Pb, S, Sr, Zn	TXRF Degasification in an ultrasonic bath	Elemental contents for all samples measured by TXRF [mg/L]: Ca: 9.82–96; Cu: <LOD–0.32 Fe: 0.07–1.57; K: 183.79–418.47 Mn: 0.06–1.42; Ni: <LOD–1.13 P: 37.40–149.85; Pb: <LOD–0.18 S: 10.32–50.73; Sr: <LOD–0.41 Zn: 0.02–1.98	[21]
73 samples (25 samples of Polish ciders, and 40 samples of low-percentage, flavored alcoholic beverages based on beer from Polish market)	Ag, Al, Ba, Ca, Cd, Co, Cr, Cu, Fe, Hg, K, Mg, Mn, Na, Ni, Pb, Sr, Ti, Zn	ICP-OES, ICP-MS, CVAAS Degasification in an ultrasonic washer + dilution with 4 mL of 65% HNO_3_ + microwave mineralization + addition internal standard solution (In) + dilution with ultrapure deionized water up to 25 mL	Metal contents for low-percentage, flavored alcoholic beverages based on beer [μg/L]: Ag: 0.046–2.569; Al: 854.4–1905 Ba: 71.58–211.2; Cd: 0.046–0.422 Co: 0.032–1.408; Cr: 2.258–74.91 Cu: 4.937–95.38; Mn: 38.29–269.0 Ni: 3.161–111.1; Pb: <LOD–16.93 Sr: 4.881–29.57; Ti: <LOD–24.02 Zn: 2.521–92.24; Hg: <LOD [mg/L]: Ca: 31.09–140.4; Fe: <LOD–1.298 K: 24.86–489.5; Mg: 21.52–114.8 Na: 17.12–334.3	[22]

**Table 2:** Basic statistics for determined elements for all beer samples (*n* = 50) [mg/L].

Elements	*n*	Concentration Unit	Mean	Median	Min	Max	Std.dev.
^55^Mn	50	µg/L	130.2	119.7	63.19	282.8	54.66
^59^Co			9.808	7.628	2.430	23.69	7.837
^60^Ni			19.68	15.48	10.20	44.67	8.703
^95^Mo			4.674	4.164	1.938	10.08	2.157
^107^Ag			3.201	2.151	<LOQ	17.73	3.596
^111^Cd			6.461	5.377	1.349	16.78	5.431
^118^Sn			1.830	1.790	0.698	4.636	0.747
^121^Sb			0.587	0.194	<LOQ	2.996	0.862
^208^Pb			12.13	11.55	<LOQ	31.34	8.383
Al 396.152		mg/L	3.125	<LOQ	<LOQ	20.06	5.786
Ba 455.403			0.032	<LOQ	<LOQ	0.837	0.124
Ca 393.366			48.16	42.45	17.14	87.75	22.41
Cr 267.716			0.044	0.041	<LOQ	0.097	0.018
Cu 327.396			0.057	0.056	<LOQ	0.095	0.016
Fe 259.940			0.280	0.203	<LOQ	0.939	0.220
K 766.490			428.9	437.4	291.3	607.5	62.27
Mg 279.553			80.19	80.28	47.33	106.4	15.19
Na 589.592			33.64	24.65	3.490	99.16	24.54
P 177.495			230.5	224.7	18.58	381.9	65.77
S 180.731			67.07	66.55	6.437	99.84	16.76
Sr 407.771			0.232	0.127	0.070	1.917	0.429
Ti 334.941			0.008	<LOQ	<LOQ	0.170	0.025
Zn 213.856			0.274	0.119	<LOQ	1.106	0.353

<LOQ–Limit of Quantification.

**Table 3:** Basic characteristics of the investigated beer samples according to type of packaging.

Brand Code	H		L		W		Z		ZZ	
									ZZB	
Type of Packaging	C	B	C	B	C	B	C	B	C	B
***n***	3	3	3	3	3	3	3	3	3	3
**Total**	6		6		6		6		6	
**Washout Experiment**	3 bottles		-		3 bottles		3 cans		3 bottles	

Abbreviations: C—can; B—bottle.

**Table 4:** Contents of selected elements (with statistically significant differences) in the measured set of samples (*n* = 30; bottles *n* = 15, cans = 15) in reference to the impact of packaging material [mg/L].

Elements	Type of Packaging	*n*	Mean	Median	Min	Max	Std.dev.
Na 589.592	bottle	15	39.93	38.12	29.69	58.74	8.877
	can		18.02	18.03	3.490	25.85	5.822
^55^Mn	bottle		94.82	93.48	46.06	136.8	32.44
	can		152.6	154.0	96.97	227.9	38.95
Al 396.152	bottle		<LOQ	<LOQ	<LOQ	<LOQ	0.000
	can		10.41	9.370	1.260	20.06	5.990

**Table 5:** Contents of Al (with statistically significant differences) in the measured set of samples (*n* = 12; bottles *n* = 9, cans = 3) in terms of the impact of packaging material, “washout” experiment [mg/L].

Elements	Type of Packaging	*n*	Mean	Median	Min	Max	Std.dev.
Al 396.152	bottle	9	<LOQ	<LOQ	<LOQ	<LOQ	0.000
	can	3	0.302	0.317	0.040	0.550	0.255

**Table 6:** The list of positively verified statistically significant differences for studied beer brands (*n* = 30).

ZZB-H	ZZB-W	ZZB-L	ZZB-Z	Z-L	Z-H	Z-W	L-H	L-W	H-W
Ag; Cd; Co	Ag; Cd; Co	Cd; Co; Mg; S; Sb; Zn; pH	pH	Cd; K; Sn; Mg; P; Zn	Ca; K; Mg; Sn; Sr	Pb	Ca; Sb	Ca; K; Mg; P; S; Sb	Sr

**Table 7:** Parameters for which there were statistically significant differences in terms of beer type (*n* = 50).

Criterion	Parameters
Lager—Ale	Ag; Cd; Co; Ni; Mn; Pb; Zn; pH
Lager—Craft	Ag; Ca; Cd; Co; Cu; Ni; Mn; Pb; Zn
Ale—Craft	pH

**Table 8:** Provisional tolerable daily intake (PTDI) for chosen elements [36,37,38,39,40,41,42,43,44,45,46].

Element	PTDI	Year	Reference
Al	0.267 mg/kg bw	2011	WHO [36]
Ba	0.200 mg/kg bw	2012	SCHER [37]
Cr	0.300 mg/kg bw	2014	EFSA [38]
Cu	0.500 mg/kg bw	1982	WHO [39]
Fe	0.800 mg/kg bw	2010	EFSA, FAO/WHO [40]
Sr	0.600 mg/kg bw	2007	US EPA [41]
Zn	0.566 mg/kg bw	2007	US EPA [41]
Ag	5.000 μg/kg bw	2007	US EPA [41]
Cd	0.833 μg/kg bw	2013	WHO [42]
Co	23.00 μg/kg bw	2012	EFSA [43]
Mn	156.0 μg/kg bw	2007	HC1 [44]
Mo	5.000 μg/kg bw	2007	US EPA [41]
Ni	13.00 μg/kg bw	2020	EFSA [45]
Sb	0.200 μg/kg bw	2014	Health Canada [46]
Sn	600.0 μg/kg bw	2007	US EPA [41]

**Table 9:** Risk assessment of chosen elements for beer samples.

No.	ICP-MS								ICP-OES						
	Mn	Co	Ni	Mo	Ag	Cd	Sn	Sb	Al	Ba	Cr	Cu	Fe	Sr	Zn
1	426	440	97.5	69.5	-	40.8	24347	116	-	-	466	1118	432	737	459
2	474	872	131	78.1	2454	57.7	39548	255	-	-	432	1022	708	702	-
3	327	110	40.7	76.0	39.5	41.4	18120	102	-	161	490	740	129	529	126
4	113	891	132	259	914	61.5	30992	253	1.86	-	1511	1113	316	576	512
5	117	1019	144	273	5260	61.1	40025	143	2.10	223	-	1216	362	483	-
6	116	1001	153	250	3291	86.4	42506	150	3.44	-	-	1390	503	575	-
7	160	1128	126	252	544	73.7	37497	11.9	-	416	476	1576	460	689	-
8	161	1325	126	213	137522	75.9	32841	11.7	-	33.4	536	1375	512	768	-
9	165	1044	119	232	323	76.6	37944	12.5	-	-	768	917	379	815	-
10	142	893	63.7	183	1195	48.4	57995	9.35	2.21	148	1689	1371	395	567	-
11	142	825	103	157	712	50.0	29635	10.2	2.42	204	1703	1101	336	505	-
12	148	826	117	164	557	49.3	37858	9.71	14.9	-	1702	1223	512	654	-
13	208	875	134	103	-	58.6	37188	107	2.90	-	1011	1391	149	601	-
14	225	810	123	107	2728	45.5	46061	115	2.34	-	879	1237	567	602	-
15	225	1008	147	106	22882	57.1	66233	194	4.17	-	999	1083	598	594	-
16	234	961	143	92.4	399	53.1	48064	122	-	-	1012	1113	650	662	-
17	287	1009	178	361	8705	59.0	61903	162	-	-	1194	1114	651	631	-
18	225	994	143	84.0	4495	56.6	66550	181	-	-	1013	1239	607	662	1314
19	107	1045	167	340	3402	58.2	59505	207	-	-	1014	1116	703	475	-
20	106	907	137	350	-	51.9	47839	168	-	-	1196	1395	588	493	-
21	101	904	121	314	1157	53.7	45868	146	-	-	1018	933	679	477	-
22	240	822	131	143	8066	51.7	38163	268	-	-	879	1391	586	530	-
23	244	851	121	167	7063	53.1	43477	217	-	-	-	-	-	531	-
24	249	1056	165	167	-	68.5	35862	-	-	-	1019	1868	571	534	-
25	291	453	155	168	264	22.5	120345	37.9	-	-	1021	1872	130	832	649
26	299	379	82.0	95.2	328	17.1	89943	24.8	-	-	703	1606	119	950	273
27	335	378	161	178	293	19.7	72492	29.5	-	-	1197	2235	123	1099	388
28	194	416	139	140	353	20.9	58051	10.4	6.35	-	1016	1096	608	780	355
29	333	387	161	189	311	18.3	79832	35.9	14.4	-	1197	1216	570	1099	2259
30	346	426	158	189	380	20.4	117420	38.6	29.7	-	1195	1214	651	1195	679
31	172	331	97.3	117	294	17.8	56199	-	-	-	1199	1399	837	829	246
32	186	418	111	125	308	21.0	73418	99.9	-	-	1193	1392	761	879	4848
33	169	334	96.0	115	129	18.5	64656	162	-	-	1195	1239	834	826	265
34	175	377	108	98.0	261	18.2	55872	129	3.99	-	1192	1391	795	734	4845
35	126	333	99.4	92.8	171	18.3	94179	113	5.91	-	1194	1393	155	778	644
36	95.9	276	57.6	317	155	15.2	56649	146	4.04	-	1199	1017	536	445	346
37	93.6	308	86.3	223	107	14.5	111252	206	-	-	1011	1011	701	776	195
38	107	297	119	352	210	16.0	113663	167	-	-	1187	852	1081	821	555
39	77.2	136	46.1	232	87.9	7.19	35419	97.5	-	-	793	947	500	452	71.6
40	99.9	154	59.9	251	102	7.86	61474	-	-	-	890	1252	771	609	116
41	102	182	62.3	354	125	8.80	74451	147	-	-	784	1248	802	514	104
42	183	135	55.7	104	91.0	7.71	43904	163	-	-	887	802	434	43.8	89.2
43	179	125	50.5	108	79.1	6.95	33187	217	-	-	780	1596	403	44.3	78.8
44	182	136	52.2	98.6	88.1	7.49	38329	142	-	-	888	1405	396	44.0	89.9
45	258	149	69.6	185	83.5	7.22	32254	204	-	-	908	885	479	1046	93.3
46	281	163	70.9	191	96.8	7.03	38098	178	-	-	897	1263	598	1033	117
47	270	142	61.3	190	82.4	6.98	30825	116	-	-	884	1018	706	1102	104
48	313	157	68.8	168	104	7.26	50096	183	-	-	1021	936	480	1021	122
49	313	149	63.4	168	97.2	7.28	40840	177	-	-	1027	1130	407	837	97.9
50	106	298	62.8	220	178	11.9	49138	129	-	-	780	860	536	828	76.4

**Table 10:** Characteristics of the investigated beer samples.

Brand code	H		L		W		Z		ZZ						C		JO		B		HM	
									ZZB		ZZJ		ZZK									
Type of Packaging	C	B	C	B	C	B	C	B	C	B	C	B	C	B	C	B	C	B	C	B	C	B
***n***	3	3	3	3	3	3	3	3	3	3	0	3	0	3	0	3	0	3	0	5	0	3
**Total**	6		6		6		6		6		3		3		3		3		5		3	
**Beer Type**	L		L		L		L		Ale		L		Ale		L		CR		CR		CR	
**Country of Origin**	Poland																					

Abbreviations: C—can; B—bottle; L—lager; CR—craft.

**Table 11:** ICP-OES (Thermo Scientific, ICAP 7400 series, Waltham, MA, USA), ICP-MS (Thermo Electron Corporation, X SERIES, Rugby, UK) parameters and measurement conditions.

Parameter and accessories	ICP OES	ICP MS
Number of replicates	3	3
Carrier gas	Argon	Argon
Plasma gas flow rate [L·min^−1^]	12	10
Auxiliary gas flow rate [L·min^−1^]	0.5	0.76
Nebulization gas flow rate [L·min^−1^]	0.5	0.9
Nebulization gas pressure [kPa]	260	290
Pump speed during the sampling [rmp]	50	30
Torch	Quartz	Quartz
Nebulizer	Concentric quartz	Concentric quartz
Generator power [W]	1150	1317
Internal standard	In	In

**Table 12:** Basic validation parameters obtained for each analyte for the developed method (*n*, number of standards in three replicates, *R*^2^, coefficient of determination, CRM’s values).

**ICP-OES**								**CV [%]**
**Analyte**	***n***	**Equation**	$R^{2}$	**LOD ** **[mg/L]**	**LOQ ** **[mg/L]**	CRM Values (TMDA 54.6, * NCS ZC 8100 2b)		
						**Certified Value**	**Obtained Value**	
Al	7	y=12195x+11.4	0.999	0.010	0.030	0.389 ± 0.028 mg/L	0.372 ± 0.001 mg/L	96
Ba	7	y=255146x+713	1.000	0.013	0.039	0.324 ± 0.019 mg/L	0.339 ± 0.001 mg/L	105
Ca	7	y=59763x+7894	1.000	0.013	0.039	* 1537 ± 68.00 μg/g	* 1592 ± 2.500 mg/L	104
Cr	7	y=19414x+421	1.000	0.006	0.018	0.421 ± 0.022 mg/L	0.428 ± 0.002 mg/L	102
Cu	7	y=19008x+318	1.000	0.006	0.018	0.393 ± 0.026 mg/L	0.392 ± 0.002 mg/L	100
Fe	7	y=12540x+78.8	1.000	0.033	0.099	0.367 ± 0.023 mg/L	0.379 ± 0.001 mg/L	103
K	7	y=444x+115	1.000	0.029	0.087	* (14.40) μg/g	* 15.84 ± 2.000 μg/g	110
Mg	7	y=23009x+216	0.999	0.081	0.243	* 248.0 ± 14.00 μg/g	* 251.6 ± 1.200 μg/g	101
Na	7	y=1593x+50.5	1.000	0.050	0.150	* 445.0 ± 40.00 μg/g	* 468.7 ± 4.500 μg/g	105
P	3	y=437x+0.50	1.000	0.012	0.036	* 174.0 ± 43.00 μg/g	* 154.8 ± 0.011μg/g	89
S	3	y=518x+15.6	0.999	0.040	0.120	* (4.620) %	* 4.361 ± 0.006%	94
Sr	7	y=41626x+38.6	1.000	0.005	0.015	0.573 ± 0.031 mg/L	0.610 ± 0.001 mg/L	106
Ti	3	y=55828x+642	1.000	0.003	0.009	0.032 ± 0.003 mg/L	0.032 ± 0.001 mg/L	100
Zn	7	y=34553x+530	0.998	0.009	0.027	0.540 ± 0.035 mg/L	0.557 + 0.006 mg/L	103
**ICP-MS**								**CV [%]**
**Analyte**	***n***	**Equation**	$R^{2}$	**LOD ** **[μg/L]**	**LOQ ** **[μg/L]**	CRM Values (TMDA 54.6, *NCS ZC 8100 2b)		
						**Certified Value**	**Obtained Value**	
Ag	7	y=646x+196	0.999	0.001	0.003	12.90 ± 1.100 μg/L	13.01 ± 0.617 μg/L	101
Cd	7	y=138x+12.7	0.999	0.001	0.003	156.0 ± 8.000 μg/L	160.3 ± 7.481 μg/L	103
Co	7	y=1047x+150	0.999	0.025	0.075	305.0 ± 20.00 μg/L	281.4 ± 0.392 μg/L	92
Mn	7	y=1036x+217	0.999	0.048	0.144	274.0 ± 13.00 μg/L	238.8 ± 1.719 μg/L	87
Mo	7	y=142x+1.96	0.999	0.039	0.117	292.0 ± 20.00 μg/L	279.7 ± 7.016 μg/L	96
Ni	7	y=222x+61.8	0.999	0.102	0.306	323.0 ± 19.00 μg/L	297.0 ± 0.366 μg/L	92
Pb	7	y=701x+44.0	0.999	0.096	0.288	490.0 ± 30.00 μg/L	473.8 ± 22.75 μg/L	97
Sb	4	y=141x+0.36	0.999	0.032	0.096	* 120.0 ± 20.00 ng/g	* 132.03 ± 15.26 ng/g	110
Sn	4	y=221x+87.9	0.999	0.002	0.006	45.30 ± 3.100 μg/L	42.53 ± 1.360 μg/L	94
Tl	7	y=1006x+45.4	0.999	0.002	0.006	28.20 ± 1.900 μg/L	28.51 ± 1.463 μg/L	101

()–reference only; * NCS ZC 8100 2b certified material used as an alternative.

## Data Availability

Not applicable.

## Sample Availability

Beer samples are available from the authors.
